# AI-assisted feedback and reflection in vocal music training: effects on metacognition and singing performance

**DOI:** 10.3389/fpsyg.2025.1598867

**Published:** 2025-08-18

**Authors:** Wen Li, Xuerong Cui, Pravina Manoharan, Lu Dai, Ke Liu, Li Huang

**Affiliations:** ^1^School of Teacher Education, Aba Teachers College, Wenchuan, Sichuan, China; ^2^School of the Arts, Universiti Sains Malaysia, Penang, Malaysia; ^3^Zhejiang Conservatory of Music, Faculty of Education, Institute of Higher Music Education, Hangzhou, China; ^4^Faculty of Education, Languages, Psychology, and Music, SEGi University, Petaling Jaya, Malaysia

**Keywords:** metacognition, AI-assisted music learning, feedback, reflection, vocal music training, pre-service teachers

## Abstract

**Introduction:**

Metacognition plays a vital role in enhancing learning outcomes and has received increasing attention in recent years. Studies have shown that accomplished musicians typically demonstrate high levels of metacognition, and that reflection and feedback are effective strategies for promoting metacognitive development. This study explores the impact of integrating artificial intelligence (AI) and e-learning tools into vocal music training. It focuses on feedback and reflection interventions aimed at enhancing the metacognitive abilities and singing performance of pre-service teachers.

**Methods:**

An experimental design was employed over a six-week training period. Participants were randomly divided into a control group (*N* = 42), which received conventional singing instruction, and an experimental group (*N* = 38), which received additional interventions comprising: (a) self-assessment through the use of an audio comparison tool, (b) dialogic feedback through interaction with a large language model (Yuanbao, Tencent’s generative AI chatbot), and (c) engagement in self-reflective journal writing. A two-way repeated measures ANOVA was employed to examine the interaction effects between time (pre-test vs. post-test) and group (experimental vs. control). In addition, linear mixed models were used to analyse the relationship between metacognitive abilities and singing performance.

**Results:**

The results demonstrated that AI-assisted training significantly affects the development of metacognitive abilities. While both the experimental and control groups exhibited significant improvements in singing performance following the intervention, no significant interaction effect between the group and time was detected. No correlation was found between metacognition and singing performance.

**Discussion:**

The significance of this study is its provision of an effective implementation framework for integrating AI and e-learning tools into music instructional practice. These technologies offer high-quality personalized feedback and foster deep reflective engagement, thereby supporting the metacognitive development process in music education contexts.

## 1 Introduction

First proposed by Flavell (1976), metacognition refers to an individual’s knowledge of and control over their cognitive processes, which Flavell later defined as “thinking about thinking” (Flavell, 1979, 1987). In the field of educational psychology, metacognition includes individuals’ awareness of their own conditions, learning plans, learning goals, and learning strategies in specific learning situations, as well as self-evaluation and adjustment in regard to the learning process (Craig et al., 2020; Schraw and Moshman, 1995). Numerous studies have shown a significant positive correlation between metacognition and learning outcomes (Choi et al., 2023; Khellab et al., 2022; Rahimirad and Shams, 2014), indicating that learners with higher levels of metacognition are more advantaged in task planning, learning strategy use, and self-regulation. Consequently, as enhancing metacognitive ability may improve academic outcomes, it is imperative that learners and educators master methods for developing metacognition and engage in the necessary training (Li, et al., 2023; Molin et al., 2020).

Research has pointed out that musicians typically have high levels of metacognition (Concina, 2019; Peynircioğlu et al., 2014), which enables them to practise more effectively. In the process of music learning, learners usually need to perform extensive self-practice independently. However, the lack of metacognition among beginner music learners makes it difficult for them to monitor the practice process effectively, resulting in inefficient self-directed learning (McPherson et al., 2019; Mieder and Bugos, 2017). There are two main reasons for this: firstly, beginners do not receive objective feedback on their self-practice, and they are unable to detect problems such as playing conditions, pitch deviations, and rhythmic instability; thus, they are unable to make corrections in a timely manner (Li et al., 2023b). Secondly, they lack the habit of reflection during practice, so they are unable to reflect on and make timely adjustments to their practice errors (Cornoldi et al., 2015; Fent et al., 2025). Bathgate et al. (2012) pointed out that if learners do not develop metacognitive skills, the effectiveness of practice is greatly reduced, leading to an inefficient cycle of blind mechanical practice.

Enhancing metacognition has become a topic of major interest in the academic community (Altıok et al., 2019; Brooks, 2022; Cer, 2019; Khellab et al., 2022; Li, 2021). Feedback and reflection are widely regarded as two of the most critical intervention strategies for enhancing metacognition (Choi et al., 2023; Fritz et al., 2020; Li et al., 2023a, 2023b; Molin et al., 2020). Feedback refers to received information or criticism that is intended to improve subsequent learning (Molin et al., 2020). Feedback plays an important role in the development of metacognition because it helps learners gain an awareness of their current state (Li et al., 2023b; Molin et al., 2020). The original meaning of “reflection” refers to the image formed on the surface of an object after light is reflected off it. In an educational context, the term refers to learners’ analysis and evaluation of their own learning processes to improve learning outcomes (Choi et al., 2023), which is an important pathway in the development of metacognition (Wu et al., 2020). Therefore, based on existing research findings, feedback and reflection can be understood as effective strategies that help learners identify problems and make improvements, while also serving as important means for developing metacognitive abilities.

With the widespread promotion of artificial intelligence (AI), the integration of music education and AI has become an irreversible trend (Dash and Agres, 2024; Yuan, 2024). The question of how to strengthen the effects of feedback and reflection with the emerging AI technology has become an urgent direction of exploration in the field of music education. AI technology provides personalised learning and offers more technological tools for teaching and learning (Carnovalini and Rodà, 2020; Chen et al., 2020; Zhai et al., 2021), especially the application of generative AI, such as ChatGPT, DeepSeek, and other models; these have also made rapid learning convenient. However, an area of research that still has not yet been explored in depth is how AI can be used to assist music learners to improve their metacognitive skills and thus their academic performance.

To examine how AI-assisted learning can foster metacognitive development in higher education music curricula, this study explored the integration of AI and e-learning tools into vocal training. Specifically, AI in this context refers to the interaction with a large language model, which provided feedback on vocal concepts and practice. Through dialogic interaction with an AI chatbot, learners received targeted practice strategies, enabling them to regulate and refine their learning approaches. Collectively, these interventions provided opportunities for self-monitoring, as learners were able to repeatedly review their recorded performances to assess their progress. Additionally, the intervention incorporated e-learning tools that enabled students to monitor their singing practice processes, compare their recordings, and engage in structured self-assessment and reflection. Therefore, this study examined the effects of AI-assisted training on enhancing both metacognition and singing performance.

### 1.1 The connection between music training and metacognition

The relationship between music training and metacognition is an emerging area of educational research. The underlying reason for this connection is the alignment between the process of musical self-practice and the core components of metacognition. Specifically, musical self-practice involves setting training goals, monitoring and evaluating practice outcomes, and continuously adjusting practice strategies (López-Íñiguez and McPherson, 2020; Mieder and Bugos, 2017). This practical approach corresponds closely with the cognitive regulation aspect of metacognition, which includes planning, monitoring, evaluating, and debugging (Craig et al., 2020; Van Loon et al., 2021).

Recent studies have emphasized the critical role of feedback in self-practice music. Li et al. (2023b) highlighted that real-time feedback is essential for effective self-monitoring and evaluation. This ongoing feedback process is a central component of efficient practice. Correspondingly, in metacognitive enhancement research, the use of real-time feedback is also a common and effective intervention strategy (Altıok et al., 2019; Molin et al., 2020). Blackwell et al. (2023) and Li et al. (2023b) pointed out that incorporating recording tools into music practice enables immediate performance assessment and encourages learners to reflect and make adjustments based on the feedback received. Moreover, activities that incorporate real-time feedback and reflection promote deeper engagement with metacognitive processes. Karaoglan Yilmaz (2022) and Molin et al. (2020) pointed out that this form of engagement enables learners to enhance their awareness of metacognitive components during music self-practice.

Finally, musical training is not merely the practice of skills; it may also involve complex cognitive processes. Neuroscientific studies by Choi et al. (2015) and Herholz and Zatorre (2012) have shown that musical training enhances various cognitive abilities, many of which are closely related to metacognition. Further research by Francisca Lupu et al. (2023) and Román-Caballero et al. (2018) indicated that the cognitive improvements brought about by music training also have a positive impact on metacognition, thereby establishing a bridge between these two domains.

In summary, this study aimed to enhance metacognition and singing performance through music training. Specifically, it incorporated AI and e-learning tools by integrating feedback and reflection-based training into the vocal instruction of pre-service teachers. This study expands the application of metacognition, feedback, and reflection theories in music education, and it summarizes effective mechanisms to enhance metacognition through music training. Accordingly, the following research questions were addressed:

- Do AI-assisted feedback and reflection in vocal music training enhance learners’ metacognition?
- Do AI-assisted feedback and reflection in vocal music training improve learners’ singing performance?
- Is there a relationship between the metacognition and the singing performance of learners?

## 2 Methods

### 2.1 Research design

**Figure 1:**
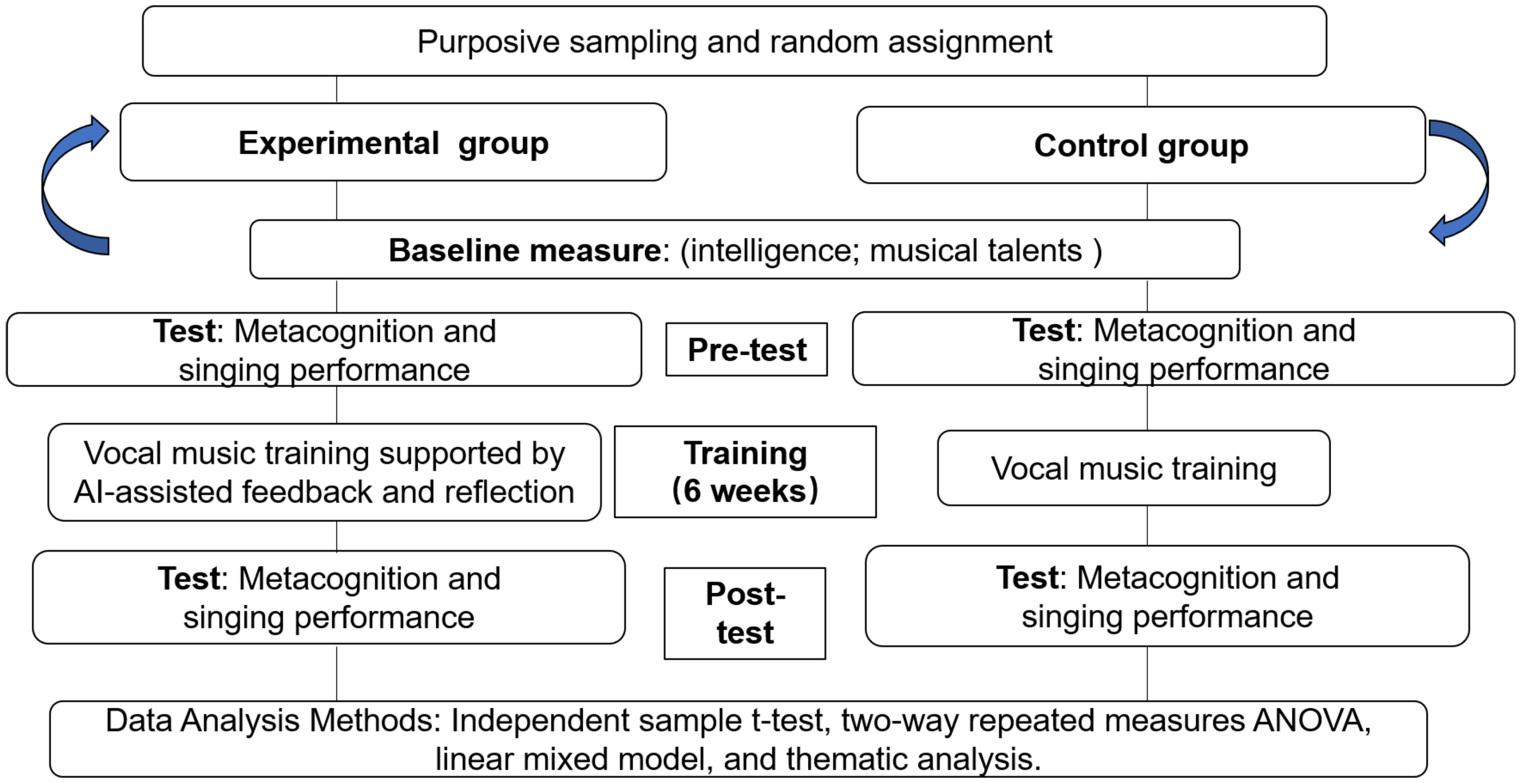
Design of the study.

This study employed a mixed-methods research approach. The quantitative component utilized an experimental design to assess how feedback and reflective practice mechanisms during vocal training affected the metacognitive abilities and singing performance of the participants. All the participants were randomly assigned to an experimental or a control group. Conducted over 6 weeks, the study employed a pre- and post-test design with experimental and control groups to compare the impact of different training methods. The qualitative component adopted a thematic analysis. Reflective journals written by pre-service teachers during vocal training were collected, as were their interaction records with AI. The qualitative findings supplemented the quantitative results. The research process comprised the following key steps (see Figure 1).

#### 2.1.1 Baseline measurement

To mitigate the impact of innate ability differences on the experimental results, a baseline measurement was conducted (White and Thompson, 2005) to measure the intelligence and musical ability of the participants. If the baseline measurements indicated significant differences between the two groups, re-randomization was performed to ensure that the participants started training at a comparable level. The intelligence test used in this study was the Stanford-Binet Intelligence Scales, Fifth Edition (Newton, 2020), while musical ability was assessed using Seashore’s Measures of Musical Talents (Seashore et al., 1956).

#### 2.1.2 Pre-test and post-test

Before the training, all participants underwent pre-testing of their metacognition and singing performance. This evaluation helped identify initial differences between groups, ensuring that subsequent measurements would accurately reflect actual improvements in ability. After the training, all the participants were reassessed for metacognition and singing performance to evaluate their progress during the training period, with a particular focus on changes in the experimental group.

### 2.2 Sampling

Purposive sampling was employed to recruit 100 pre-service teachers from a teacher education college in Guizhou Province, China. There were two reasons for selecting pre-service teachers as the sample population. First, these teachers are at a critical stage of the development of their teaching styles, making it essential for them to enhance metacognitive skills that enable them to reflect on their teaching practices (Yokuş, 2021). Second, pre-service teachers typically possess a foundational background in vocal training (Yang and Welch, 2023), which aligned with this study’s focus on improving singing performance.

The sample size of this study was calculated using G*Power 3.1, based on two-way repeated measures ANOVA (Within-Between Interaction). The study parameters were determined as follows: an effect size of 0.25 was chosen to allow for the detection of even modest effects, such as subtle gains resulting from the intervention. According to the Institute of Education Sciences (IES) in the United States, an effect size of 0.25 is considered the threshold for a finding to be regarded as ‘substantively important’ (Cohen, 2013; Simpson, 2020). In addition, the significance level (*α*) was set at 0.05, statistical power was set at 0.95, and correlation among repeated measures was set at 0.5 (Faul et al., 2009). Two measurements were taken (pre-test and post-test), with two groups (experimental and control). The calculation indicated that a total sample size of 54 participants (27 per group) would be required. In this research, a total of 100 pre-service teachers were recruited prior to the commencement of training. Participants were randomly assigned to either the experimental (*n* = 50) or the control group (*n* = 50), ensuring equal group sizes. However, data from only 80 participants were ultimately included in the analysis due to scheduling conflicts and the failure of some participants to complete the weekly vocal practice tasks or submit their recordings and reflective journals. The final sample comprised 38 participants in the experimental group and 42 in the control group, exceeding the minimum requirement of 27 participants per group.

The participants were between 18 and 21 years old. The experimental group had a mean age of 19.47 (SD = 0.21) and consisted of 33 females and five males, while the control group had a mean age of 18.91 (SD = 0.34), with 36 females and six males in this group. This study received substantial support from the participants’ institutions. All the participants voluntarily participated and signed electronic consent forms, and they were informed of their right to withdraw from the study at any time during the training. Additionally, as an incentive for full participation, an agreement was reached with the institution to award students who successfully completed the training an extra 5–10 points on their final music examination.

In addition, the researchers and three female vocal instructors participated in the training process. Vocal instruction was delivered in a small-group format, with five students per group, meeting once a week for 1 over a period of 6 weeks. The researcher provided a single 60-min session to both the instructors and the experimental-group participants, during which metacognitive theory was explained and e-learning tools were introduced. Participants in both the experimental and the control groups were randomly assigned to one of three vocal instructors.

The three vocal instructors jointly undertook the vocal instruction tasks for both the experimental and the control groups. Each instructor had over a decade of experience in vocal training, and their ages ranged from 32 to 40 years. The instructors followed the Zhou Xiaoyan Vocal Teaching System (周小燕声乐教学体系), ensuring consistent teaching content and methods. Professor Zhou Xiaoyan is a renowned vocal music educator in China. She has published multiple sets of vocal music textbooks and video courses, gaining widespread recognition nationwide (Tu, 2024).

### 2.3 Training process

The training process in this experiment consisted of three main components, listed as follows:

Part 1: self-assessment through audio comparison

**Figure 2:**
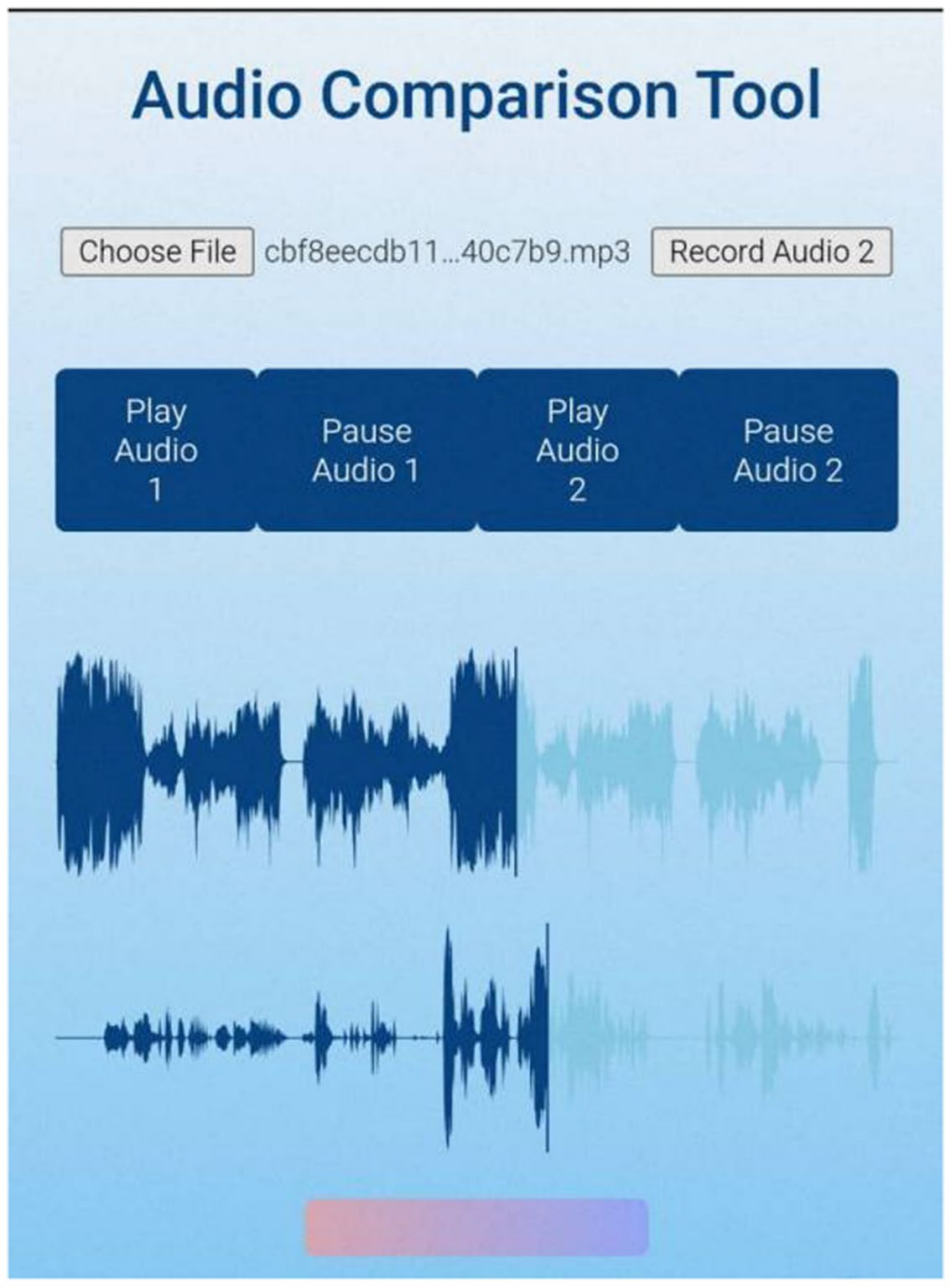
Operation interface of the audio comparison tool.

In the first part, students engaged in self-assessment of their practice using an audio comparison tool. In this study, generative AI was employed to generate code in order to develop the audio comparison tool used in music practice (for further details on the development process, see the Research Instruments section). This programme featured both recording and audio comparison functions, allowing users to record their voices by clicking a button. After recording, users could compare their newly recorded audio with a previously uploaded teacher’s demonstration recording. Figure 2 illustrates the programme interface, which supported dual audio playback and pause functions.

The key feature of this tool was its ability to allow students to compare their singing with the demonstration performance, helping them to identify discrepancies in tone quality, pitch, and rhythm. Additionally, students could observe audio waveforms to compare differences visually. Through this process, learners conducted self-assessments and gained insights into their strengths and areas for improvement in singing, which served as a reference for subsequent practice (see Figure 2).

Part 2: AI-based personalized dialogic feedback

**Figure 3:**
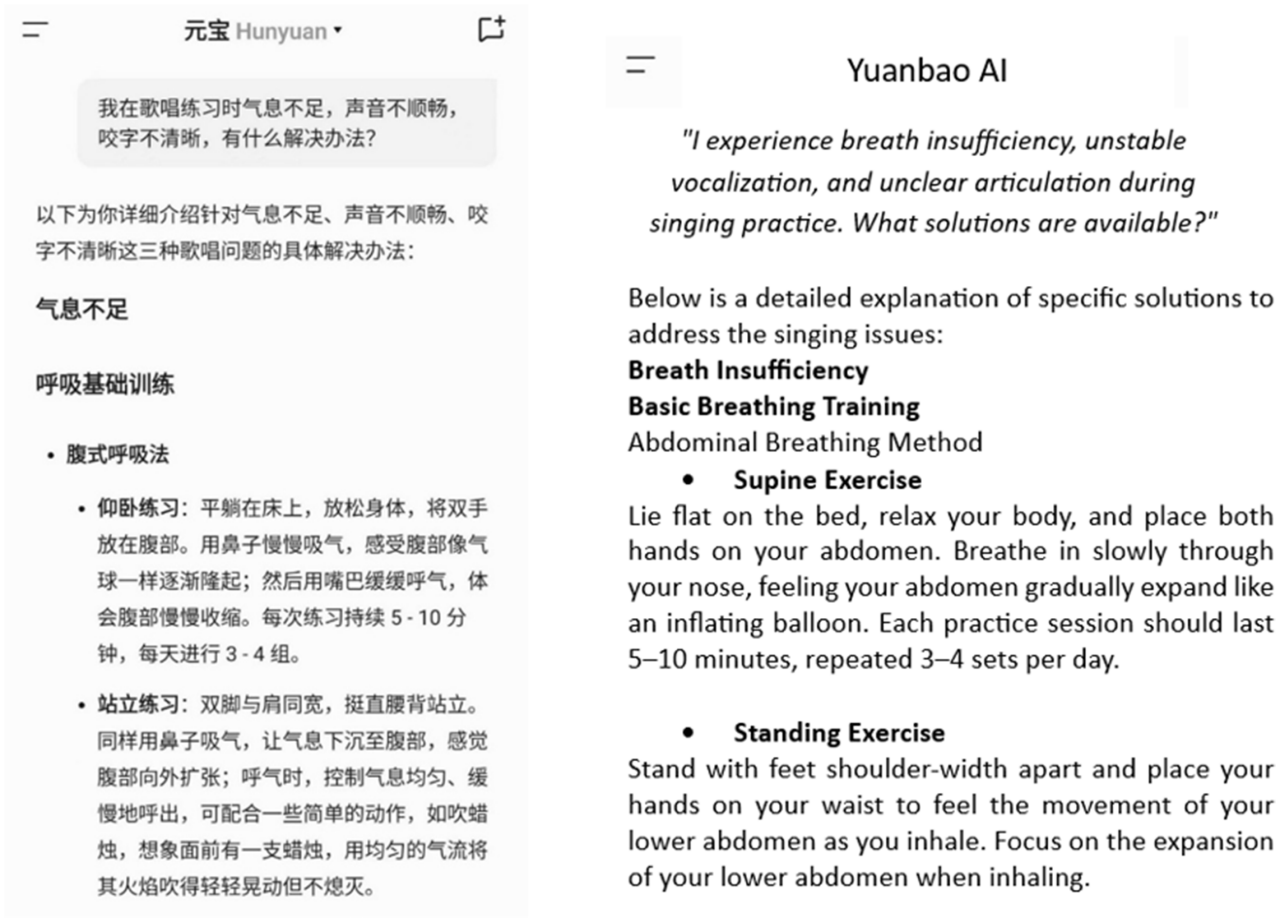
AI-dialogic feedback for singing practice.

In the second training phase, the students utilised Tencent’s Yuanbao AI (for details, see the Research Instruments section). Participants were able to input specific issues encountered during their practice into the AI and obtain targeted adjustment strategies through dialogic feedback (see Figure 3). These strategies encompassed various aspects, including practice methods, vocal breathing control, pitch deviation correction, and rhythm adjustment, thereby facilitating the continuous refinement and optimization of practice routines.

Part 3: submission of practice recordings and reflective journals

**Figure 4:**
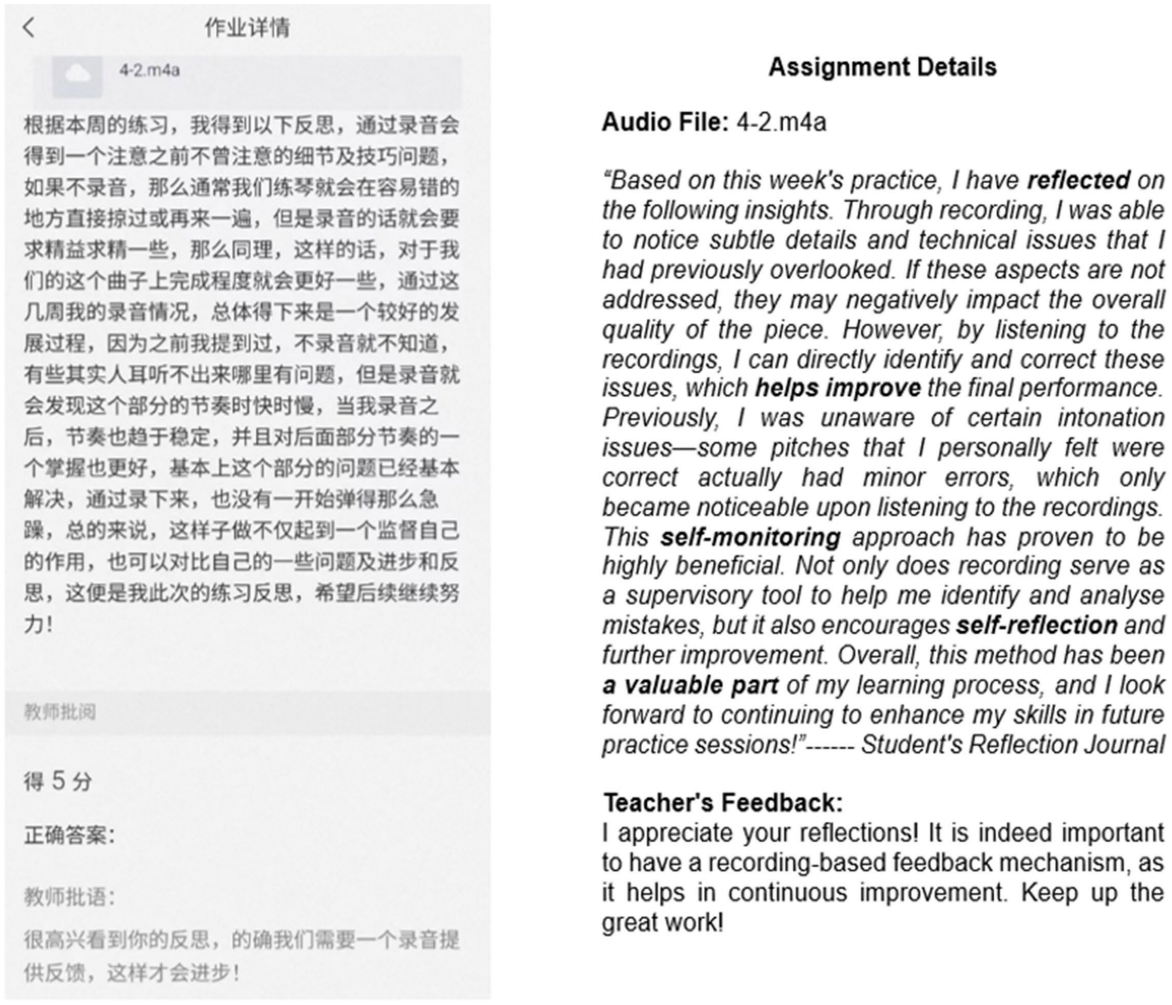
Xuexitong E-learning platform.

Students were required to use the Xuexitong e-learning application to upload at least four practice recordings per week and to write reflective journals about their weekly practice (see Figure 4). This approach aimed to monitor students’ progress and ensure consistency. By documenting their achievements and challenges, students engaged in self-reflection, enabling them to make adjustments in subsequent practice sessions and fostering the development of metacognitive skills.

The screenshot in Figure 4 shows a student’s reflection journal after a singing practice session.

Both the experimental and control groups were required to practice four times per week and submit their weekly self-practice recordings. Throughout the training period, the control group did not utilize the audio comparison tool, write reflective journals, or receive dialogic feedback from the AI. In addition, the experimental group also received feedback from instructors based on their submitted audio files. It is important to note that the instructors’ feedback focused on identifying issues in the recordings and offering affirmation and encouragement to the participants, without directly providing solutions to the singing problems. The intention of design was to encourage participants to engage in self-regulation practice and enhance their metacognitive abilities through the use of an audio comparison tool, AI dialogic feedback model, and reflective journals.

### 2.4 Research instruments

#### 2.4.1 Audio comparison tool

An audio comparison tool was developed in this study to support the vocal training of the pre-service teachers (see Figure 2). Prior to its development, a needs analysis was conducted to identify four core functions for the tool: the ability to upload multiple audio files, real-time playback, recording, and the visualization of audio differences. To ensure the tool would efficiently implement the audio comparison function, relevant code was generated with the assistance of the generative AI software ChatGPT-4o. Based on JavaScript and the Web Audio API, the code utilized the Wavesurfer.js library to visualize audio waveforms and compare differences between two recordings. Code editing and testing were performed online via CodePen, which provided a visual editing environment and real-time previews of the audio playback and comparison results. The researcher subsequently refined the original code to optimize the audio loading, playback/pause functionality, and visualization of differences. To deploy the full functionality of the application, the tool was hosted on GitHub and made accessible online via GitHub Pages. For the user interface of the audio comparison tool, see the following website: https://coco840.github.io/LW/.

#### 2.4.2 Yuanbao AI

Tencent’s Yuanbao AI system was employed to deliver personalized dialogic feedback in the context of music learning. In this setting, learners could input specific issues encountered during their singing practice and receive targeted adjustment strategies from the AI (see Figure 3). Yuanbao is an AI assistant application launched by Tencent on May 30, 2024, whose operation is similar to ChatGPT. The application offers various services, including text generation using natural language processing, AI-assisted image creation, and voice-based interactions.

#### 2.4.3 Xuexitong E-learning tool

Xuexitong is a widely used e-learning platform in China that enables instructors to upload course syllabi, share learning materials, assign homework, conduct online examinations, and facilitate community discussion. In this study, the platform was used for homework submission. Instructors reviewed the students’ weekly practice recordings and reflective journals submitted through the platform to monitor their learning progress (see Figure 4).

#### 2.4.4 Metacognition scale for music learning

The Metacognition Scale for Music Learning (Li et al., 2023a) was used to collect data on the participants’ metacognitive levels in music learning during both the pre- and post-test phases. Developed based on the Metacognitive Awareness Inventory (Schraw and Dennison, 1994), the scale was modified for applicability in the music domain. The instrument employs a five-point Likert scale and consists of 35 items divided into two sections—music metacognitive knowledge and music metacognitive regulation—encompassing eight factors: music declarative knowledge, music procedural knowledge, music conditional knowledge, planning, self-assessment of musical ability, information management, monitoring, evaluation and debugging (Li et al., 2023a). The high reliability of the scale used in this study was indicated by its Cronbach’s alpha value of 0.97.

#### 2.4.5 Stanford-Binet intelligence scales, and Seashore measures of musical talents scales

Two scales were used in the baseline measurement to assess the intelligence and musical ability of the pre-service teachers. The Stanford-Binet Intelligence (SBI) scales (Newton, 2020) comprise 29 items that evaluate four aspects— quantitative reasoning, knowledge, visual-space, and memory—and have a reliability (Cronbach’s alpha) of 0.92. The 1956 edition of the Seashore Measures of Musical Talents (SMMT) (Seashore et al., 1956), revised by Carl E. Seashore in collaboration with Lewis and Saetveit, provides a comprehensive assessment of six core musical aptitudes: pitch, intensity, rhythm, time, timbre, and tonal memory. This edition aimed to provide a more standardized and modernized tool for assessing musical abilities. Compared to other musical assessment tools, the Seashore test offers an objective, laboratory-based standard for evaluating musical talent. It has made major contributions to the standardization, accessibility, and cost-effectiveness of musical ability measurements (Devaney, 2019).

#### 2.4.6 Auditory-perceptual rating instrument for the operatic singing voice scale

The Auditory-Perceptual Rating Instrument for the Operatic Singing Voice Scale (APRIOSV) scale (Oates et al., 2006), was used to assess the singing performance of the pre-service teachers. Five dimensions from the APRIOSV scale were selected for this study: ring, pitch, breath management, evenness throughout the range, and strain. Given that the original APRIOSV scale was designed for evaluating operatic singing, the selection of these specific dimensions was deemed appropriate for assessing the performance of general vocal learners. The reliability Cronbach’s alpha value is 0.97.

Two vocal music evaluators, each with over 10 years of experience in vocal performance and teaching, were invited to evaluate the singing recordings of the pre-service teachers. These evaluators were independent of the instructors who conducted the vocal training for this study.

As noted by Kreiman et al. (2007) and Merrill (2023), perceptual voice evaluation often yields limited interrater reliability. To enhance consistency in the assessment process, a calibration session was conducted among the evaluators prior to the formal evaluation. During this session, the evaluators were introduced to the APRIOSV scale, and the scoring criteria were explained. This process ensured that both evaluators developed a shared understanding of the rating standards. To assess inter-rater reliability, the intraclass correlation coefficient (ICC) was calculated using a two-way random effects model with a definition of consistency. The average-measure ICC was 0.89 (95% CI: 0.82–0.93), indicating a high level of agreement between the two vocal instructors. The participants performed the same singing piece before and after training. The selected piece was drawn from the repertoire studied during vocal lessons.

### 2.5 Data analysis

The metacognition score for each participant was obtained by calculating the mean of their responses to all 35 items on the Metacognition Scale for Music Learning, with each item rated on a 5-point Likert scale ranging from 1 (strongly disagree) to 5 (strongly agree). The singing performance scores were evaluated by two trained vocal instructors using the APRIOSV singing assessment scale. The assessment comprised five dimensions: ring, pitch, breath management, evenness throughout the range, and strain. Each participant was rated by both evaluators, and the final score was calculated as the average of the summed ratings across these five dimensions.

In this study, data analysis was conducted using two-way repeated measures ANOVA to examine the effects of training (experimental vs. control groups) and time (pre-test vs. post-test) on two dependent variables: metacognition scores and singing performance scores. This analysis assessed main effects (group and time) and their interaction to determine overall improvement and whether it differed between groups (Boisgontier and Cheval, 2016). In addition, an independent samples *t*-test was used to compare the differences between the two groups in the pre-test (Ross and Willson, 2017).

To examine the extent to which changes in metacognitive ability could predict singing performance, a linear mixed model (LMM) analysis was conducted using SPSS version 26.0. The LMM approach allows for the inclusion of both fixed and random effects and is well-suited for handling data with grouped structures and repeated measurements (Bolker, 2015). This analysis incorporated post-test singing performance as the dependent variable, with pre-test metacognitive scores and pre-test singing performance scores entered as fixed-effect covariates to control for baseline differences.

The model included random intercepts for group to account for group-level variability. The linear mixed model was specified as follows:

$Y_{\mathrm{ij}}=\beta_{0}+\beta_{1}X_{\mathrm{ij}}+\sum_{k}\eta_{k}C_{\mathrm{kij}}+\mu_{j}+\varepsilon_{\mathrm{ij}}$

In the model, *Y_ij_* represents the post-test singing performance score of individual *i* in group *j*, *X_ij_* denotes the metacognitive ability of individual *i* in group *j*, *C_kij_* represents the *k* covariate, where *k* = 1 indicates pre-test singing performance and *k* = 2 indicates metacognitive pre-test score, $\mu_{j}$ is the random effect associated with group *j*, and $\varepsilon_{\mathrm{ij}}$ is the residual error term.

Prior to data analysis, normality and homogeneity of variance tests were conducted to ensure that the assumptions for repeated measures ANOVA and LMM were met. In the data analysis process, *p* < 0.05 was adopted as the threshold of statistical significance. To ensure the reliability of the analysis, partial eta-squared ( $\eta_{p}^{2}$ ) values were reported to indicate effect sizes, providing insights into the magnitude of the observed effects. Cohen (2013) emphasized the importance of reporting effect sizes and practical significance, rather than solely relying on *p*-values.

Regarding the qualitative data analysis, thematic analysis was employed to examine the reflective journals. A three-stage coding approach was utilized (Creswell and Poth, 2016). First, open coding was performed on the raw data to extract content relevant to the research questions. In the second step, axial coding built upon the open coding by extracting key information and organizing it into sub-themes. Finally, sub-themes were consolidated into overarching themes (Richards and Hemphill, 2018). To facilitate analysis, each reflective journal content was coded using a unique identifier. For example, CW-2 includes an abbreviated name of one of the pre-service teachers, and the item was collected from Week 2 of the training.

## 3 Results

### 3.1 Baseline comparisons between groups

Prior to training, baseline measurements were conducted on the experimental and control groups to ensure there were no significant differences in terms of intelligence and musical talent. An independent-sample *t*-test was employed. As Table 1 illustrates, there was no significant difference in intelligence between the experimental and control groups, *t* (78) = 0.34, *p* = 0.20. Similarly, there was no significant difference in musical talent between the groups, *t* (78) = 0.65, *p* = 0.52. Thus, the requirements for experimental intervention were satisfied.

**Table 1:** Baseline measurements of Stanford-Binet intelligence scales and seashore musical talent scales.

Assessment	Group	M	SD	*t*	*p*	*df*
Stanford-Binet intelligence scales	Experimental (*N* = 38)	17.53	2.98	0.34	0.20	78
	Control (*N* = 42)	17.29	3.35			
Seashore musical talent	Experimental (*N* = 38)	20.18	4.11	0.65	0.52	78
	Control (*N* = 42)	19.60	4.32			

Descriptive statistics were produced for both the experimental and control groups on their metacognition scores and singing performance across the pre- and post-tests, as presented in Table 2. In addition, box plots were generated to illustrate the distribution of the scores and identify potential outliers, as shown in Appendix.

**Table 2:** Mean values and standard deviations for metacognition and in both groups.

Variable	Control group (*n* = 42)		Experimental group (*n* = 38)	
	Pre-test	Post-test	Pre-test	Post-test
	Mean value (standard deviation)		Mean value (standard deviation)	
Metacognition	3.52 (0.46)	3.46 (0.37)	3.44 (0.37)	3.69 (0.46)
Singing performance	3.07 (0.73)	3.31 (0.71)	3.25 (0.69)	3.62 (0.78)

### 3.2 Effects of the intervention on metacognition

An independent samples *t*-test was conducted to examine whether there was a significant difference between the experimental and control groups in terms of the pre-test metacognition scores. The results showed that the difference between the two groups was not statistically significant, *t* = 0.33, *p* = 0.74, indicating that the groups were comparable.

Repeated measures ANOVA was conducted to examine the interaction effect between training (experimental vs. control groups) and time (pre-test vs. post-test) on the metacognition scores. The analysis revealed that the interaction effect between group and time was statistically significant, *F*_(1, 78)_ = 5.10, *p* = 0.03, $\eta_{p}^{2}$ = 0.06, indicating that the improvement in metacognition scores over time differed between the two groups (Table 3). However, the main effect of time was not statistically significant, *F*_(1, 78)_ = 2.02, *p* = 0.16, $\eta_{p}^{2}$ = 0.01. Similarly, the main effect of group was not statistically significant, *F*_(1, 78)_ = 1.44*, p* = 0.23, $\eta_{p}^{2}$ = 0.02. This result suggests that training had a differential effect on the metacognition scores.

**Table 3:** Repeated measures ANOVA results for metacognition scores.

Effect	*SS* (Effect)	*SS* (Error)	*F*	*p*	Partial *η*^2^
Group	0.24	13.15	144	0.23	0.02
Time	0.36	13.98	2.02	0.16	0.03
Group× Time	0.92	13.98	5.10	0.03	0.06

**Figure 5:**
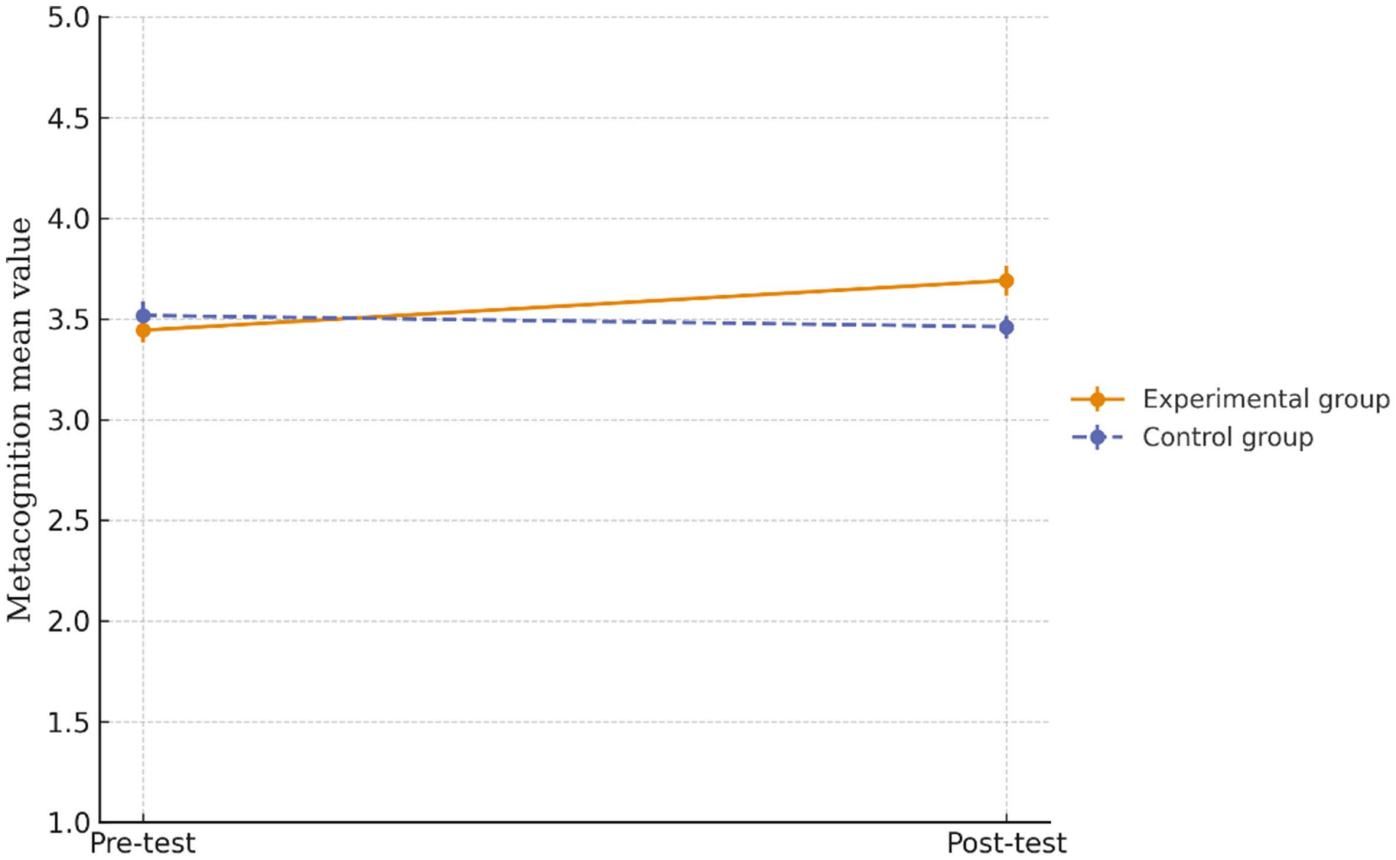
Mean metacognition scores at pre-test and post-test for experimental and control groups. Error bars represent ± 1 standard error of the mean.

Figure 5 illustrates the comparison of the mean metacognition scores obtained by the experimental and control groups at two time points: pre-test and post-test. As shown in Figure 5, the experimental group demonstrated an increase in metacognition scores, which rose from 3.44 to 3.69. In contrast, the control group showed a slight decrease in metacognition scores, which declined from 3.52 to 3.46 points. This divergent trend suggests that the experimental group’s training may have effectively enhanced the metacognitive skills of these participants.

### 3.3 Effects of the intervention on singing performance

Independent samples *t*-tests were conducted to compare the singing performance scores obtained by the experimental and control groups in the pre-test. The results indicated no significant difference between the two groups before training (*t* = 1.15*, p* = 0.25), with the control group having an average score of 3.07 and the experimental group having an average score of 3.25. Although the experimental group’s score was slightly higher, the difference was insufficient to indicate a notable disparity at baseline.

Repeated measures ANOVA was conducted to analyse the interaction effect between training (experimental vs. control groups) and time (pre-test vs. post-test) on the singing performance scores. The analysis revealed that the main effect of time was statistically significant, *F*_(1, 78)_ = 57.88, *p* < 0.001, $\eta_{p}^{2}$ = 0.43, indicating that singing performance improved between the pre- and post-tests across all participants. However, the main effect of group was not statistically significant, *F*_(1, 78)_ = 2.45, *p* = 0.12, $\eta_{p}^{2}$ = 0.03, suggesting no significant difference in singing performance between the experimental and control groups. Additionally, the interaction effect between group and time was not statistically significant, *F*_(1, 78)_ = 2.38, *p* = 0.13, $\eta_{p}^{2}$ = 0.03, indicating that the improvement in singing performance over time did not differ significantly between the two groups (see Table 4).

**Table 4:** Repeated measures ANOVA results for singing performance scores.

Effect	*SS* (Effect)	*SS* (Error)	*F*	*p*	Partial *η*^2^
Group	2.42	77.24	2.45	0.12	0.03
Time	3.85	5.19	57.88	0.00	0.43
Group × Time	0.16	5.19	2.38	0.13	0.03

**Figure 6:**
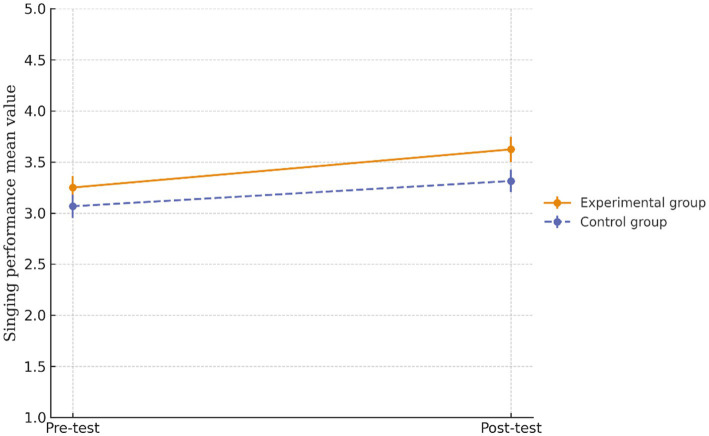
Mean singing performance scores at pre-test and post-test for experimental and control groups. Error bars represent ± 1 standard error of the mean.

Figure 6 illustrates the comparison of the mean singing performance scores between the experimental and control groups at two time points: pre-test and post-test. Both groups demonstrated improvements in singing performance over time. Specifically, the experimental group’s mean score increased from 3.25 to 3.62, while that of the control group increased from 3.07 to 3.31.

### 3.4 Regression analysis of metacognition and singing performance

The linear mixed model analysis revealed that, after controlling for pre-test singing performance and pre-test metacognitive scores, post-test metacognition did not significantly predict post-test singing performance, *t* = 0.23, *p* = 0.82. As shown in Table 5, the linear mixed-effects model yielded a marginal R-squared value of 0.76, indicating that the fixed effects—including metacognition and pre-test scores—explained approximately 76% of the variance in singing performance.

**Table 5:** Results of linear mixed model analysis.

Predictor variable	Estimate	*t*-value	*p*-value
Intercept	0.74 (0.57)	1.30	0.20
Post-test metacognition	0.02 (0.10)	0.23	0.82
Pre-test singing	Fixed		
Pre-test metacognition	Fixed		
Group (random effect)	Random		
R-squared	0.76		

Standard errors are reported in parentheses following the estimates.

### 3.5 Thematic analysis of reflection journals

Reflective journals were collected from the participants in the experimental group. As shown in Table 6, through thematic analysis of the collected data, three themes were identified: (1) the role of comparison in enhancing self-reflection, (2) the impact of AI dialogic feedback on refining practice strategies, and (3) the development of metacognition through continuous reflection. Each theme was further divided into sub-themes.

**Table 6:** Themes and sub-themes regarding participants’ reflective journals.

Themes	Sub-themes
Theme 1: The role of comparison in enhancing self-reflection	Monitoring and self-assessment of self-practice. Fosters motivation for continuous improvement. Recording Renders Hidden Issues
Theme 2: The impact of AI dialogic feedback on refining practice strategies	Multi-turn dialogic with generative AI helps clarify technical difficulties. Under AI guidance, targeted and actionable practice plans are developed. Integrating AI dialogic with teacher feedback continually optimises practice approaches.
Theme 3: The development of metacognition through continuous reflection	Self-monitoring and problem identification. Self-regulation and strategy optimisation. Self-evaluation and increased learning motivation.

#### 3.5.1 Theme 1: the role of comparison in enhancing self-reflection

This theme focuses on how participants engaged in recording their own practice sessions and comparing them with teacher-provided model performances, which helped them to identify issues in their learning more clearly, fostered motivation for continuous improvement, and prompted deeper self-reflection. Participants’ reflections illustrate these developments as follows:

*Sub-theme 1: monitoring and self-assessment of self-practice*

- By recording, I felt that I would pay attention to little details and techniques that I would have overlooked before… [I] often compared the teacher’s recording with my own to monitor my progress and check whether I had improved.” (XY-1) (Monitoring)
- After reviewing my first recording, I realized that without a clear standard to compare against, it’s easy to assume you are performing correctly. But once I had that reference, I started to recognize inaccuracies and kept correcting myself. (CW-2) (Self-assessment)

*Sub-theme 2: fosters motivation for continuous improvement*

- Compared to the earlier aimless practice, the current approach introduces comparability, allowing me to clearly see my learning outcomes, which in turn increases my motivation to continue practising.” (XY-2) (Motivation)
- In contrast to the feeling of uploading the recordings today, I feel that there is still more progress, [I’m] getting more and more proficient… compared to the previous recordings, [this] has been much better. (PJ-3) (Improvement)

*Sub-theme 3: recording renders hidden issues*

- I do not know the problem without recording, recording reveals that the tempo of this part is sometimes fast and sometimes slow… When I recorded it, the tempo also stabilized. (XY-4) (Hidden Issues)
- I originally thought I was very proficient, but recording always reveals new problems, striving for perfection… Without recording, I would not have pursued a completeness. (LY-6)

#### 3.5.2 Theme 2: the impact of AI dialogic feedback on refining practice strategies

When participants encountered practice bottlenecks, they obtained practice strategies and methods by asking questions to the generative AI. By combining this approach with professional feedback from the teacher, they continuously adjusted their practice methods to form a better practice programme. The specific participant reflections were as follows:

*Sub-theme 1: multi-turn dialogic with generative AI aids in clarifying technical difficulties*

- When I practise, my teacher always says that my breath is lacking and unclear articulation. But I do not know why this is the case and what exactly I can do to improve. So I asked the AI: “I do not have enough breath for my singing practice, my voice is not smooth and my articulation is not clear, what is the solution?” It gave me a list of abdominal breathing techniques using the supine exercise: lie flat on your back, relax your body, and place your hands on your abdomen. Inhale slowly through your nose and feel your abdomen gradually bulge like a balloon. Each exercise lasts 5–10 min and is performed in 3–4 sets per day. I find these methods very useful for me. (TY-2) (Clarifying Technical Difficulties)
- I have been having problems with throat tension during practice, and when I asked AI, I learnt that I could use the humming exercise methods. These methods are consistent with those taught by my teacher and have given me the confidence to practise and understand that it is my own lack of perseverance in daily practice that causes the problem. (ZL-3)

*Sub-theme 2: under AI guidance, targeted and actionable practice plans are formulated*

- I always feel like I cannot get my soprano voice up when I’m practising my voice. [I] asked AI: “How to train vocal soprano singing?” AI told me to first master the correct chest-abdominal joint breathing training, do more training in the middle voice area, and finally gradually raise the pitch, and pay attention to the opening of the mouth when singing soprano. The whole practice process is like a checklist, as there are specific steps that keep me… organized. (ZD-3)

*Sub-theme 3: Integrating AI dialogic with teacher feedback continually optimizes practice strategies*

- At first, I found that the practice methods suggested by the AI differed somewhat from my teacher’s instructions. For instance, the AI recommended opening the mouth when singing high notes, whereas my teacher advised using a sensation similar to yawning. Later, I combined the two methods by opening my mouth while simulating a yawn, and I discovered that my practice improved considerably. (ZD-4) (Integrating AI Dialogic with Teacher Feedback)
- After every class, if I encountered any unresolved issues, I would ask the AI again, especially those key points emphasised by the teacher that I did not fully understand. Every time, the AI was able to explain the teacher’s points in simple language, and I felt that using the AI accelerated my progress. (HZ-6) (Optimises Practice Strategies)

#### 3.5.3 Theme 3: the development of metacognition through continuous reflection

This theme focuses on how students continuously improve their self-monitoring, self-regulation, and self-evaluation abilities through various practices in music training—such as recording, self-reflection, comparison, and adjustment of practice strategies—thereby further enhancing their metacognition.

*Sub-theme 1: self-monitoring and problem identification*

- By way of recording, I would pay attention to small details and techniques that I had previously overlooked… I would also compare the previous recordings to see if I had improved in any way. (ZL-1) (Self-monitoring)
- Because, here, this rhythm I cannot get right, but I did not realize it myself… After recording it… I realised the problem. (CW-2) (Self-monitoring)
- Without the recording, I would not be able to identify the problems in my practice. Sometimes it’s not actually audible to the human ear… Recording reveals the problem with this part. (YL-3) (Problem Identification)

*Sub-theme 2: self-regulation and strategy optimization*

- In the subsequent sessions, I consistently compared my recordings during practice. identifying issues and continuously making corrections. Through the four practice sessions this week, I have essentially mastered the challenging aspects of this piece. (QL-6) (Self-regulation)
- Slow practice refers to deliberately reducing the overall tempo in order to examine the piece more carefully… It is not about the duration, but about focused attention and careful execution. (HZ-3) (Strategy Optimization)

*Sub-theme 3: self-evaluation and enhancement of learning motivation*

- Transitioning from aimless practice to having comparable recordings allows us to see whether our progress reflects improvement, stagnation, or regression. (XY-6) (Self-evaluation)
- Persistent practice enables me to identify and understand my strengths and weaknesses, allowing for continuous optimization throughout the process. It is a long-term endeavor, and I hope to continue this method indefinitely. (LY-6) (Enhancement of Learning Motivation)
- “During practice, I have learned to think critically—listening repeatedly to my own singing [and] reflecting deeply on it—because without such reflection, improvement is unlikely. I intend to continue using this method in the future. I am grateful to my teacher for this guidance! (ZD-5) (Enhancement of Learning Motivation)

In summary, the analysis of the reflection journals indicated that participants benefited from using the audio comparison tool during vocal practice. By repeatedly listening to their recordings, they were able to identify previously unnoticed issues, which fostered the development of self-monitoring and self-evaluation habits, while also enhancing their learning motivation.

Additionally, AI-based dialogic feedback helped learners clarify specific technical issues encountered during practice and provided concrete, actionable solutions. The integration of AI feedback with teacher guidance further optimized their practice strategies.

Most importantly, the analysis revealed a strengthening of the key components of metacognition, indicating overall improvement in metacognition. The combined use of AI-assisted feedback and reflective journaling not only supported the enhancement of singing performance but also significantly improved learners’ metacognitive abilities.

### 3.6 Follow-up

As a subsequent step in the research, follow-up interviews were conducted with the three instructors involved in the vocal music training to systematically examine the strengths and weaknesses of the training program. The interviews focused on instructors’ perceptions of changes in learners. Qualitative analysis of the interview data revealed that the training led to noticeable improvements in the pre-service teachers’ comprehension. However, some challenges remain, including limited interaction and a weak foundation in vocal knowledge, which affected the effectiveness of the program.

*Positive statements*:

With regard to implementation outcomes, the instructors noted:

- The students made progress in every class; they gradually learned to self-adjust, and their comprehension also improved. (LM)

Regarding improvements in learning methods, the instructors stated:

- At the beginning, the students had difficulty understanding vocal concepts. After the training—especially when using AI to ask questions and resolve their doubts—they gradually came to understand the instructional intentions. (LZG)

*Negative statements:*

One major concern raised by instructors was insufficient interaction. As one instructor noted:

- Vocal music learning emphasises the importance of feedback and real-time interaction between teachers and students. Can AI effectively support a personalized learning process and replace teachers’ roles? In my view, it is still uncertain. (TW)

The singing abilities and training period limited the effectiveness of the training implementation.

- Some students had weak vocal music knowledge and skills, and [they] often could not understand even the most basic concepts. (LZG)
- Within the limited training period, it was difficult to observe improvements in singing performance. (TW)

## 4 Discussion

### 4.1 The relationship between metacognition and singing performance

A significant interaction effect between group and time was observed for metacognition [*F*_(1, 78)_ = 5.10, *p* = 0.03], with the experimental group showing greater improvement compared to the control group. This indicates that the integration of AI-assisted feedback and reflective strategies was effective in enhancing metacognitive development among pre-service teachers.

Regarding singing performance, both the experimental and control groups showed significant improvements from pre- to post-test, as evidenced by a significant main effect of time [*F*_(1, 78)_ = 57.88, *p* < 0.001], the interaction effect between group and time was not statistically significant [*F*_(1, 78)_ = 2.38, *p* = 0.13]. This suggests that traditional instruction and the approach incorporating AI and e-learning tools were both effective in enhancing singing performance.

Moreover, the results of the LMM analysis indicated that, after controlling for pre-test singing performance and pre-test metacognitive levels, no correlation was found between metacognition and singing performance. (*t* = 0.23, *p* = 0.82). Notably, empirical research examining the specific relationship between metacognition and music performance remains limited. This study contributes to addressing that gap to some extent.

This finding further explains that the influence of metacognition on musical performance may be indirect. Although previous studies have suggested that the use of metacognitive strategies can positively impact learning outcomes (Choi et al., 2023; Khellab et al., 2022; Rahimirad and Shams, 2014). However, a meta-analysis of 118 studies examining the link between metacognition and academic achievement revealed that the impact of metacognition was not straightforward. Specifically, the association became evident only when intelligence was included as a control variable (Ohtani and Hisasaka, 2018). Additionally, as noted by the instructors during the interviews, the participantsin in this study generally lacked vocal music knowledge. Consequently, a longer period of instruction and practice may be required for such learners to effectively apply metacognitive strategies to enhance their singing performance.

### 4.2 Mechanism of metacognition enhancement through feedback and reflection

This study proposes a multi-method integrated approach to enhancing metacognitive development. Specifically, the approach consists of: (a) self-assessment using an audio comparison tool, (b) dialogic feedback through interaction with a large language model (Yuanbao, Tencent’s generative AI chatbot), and (c) engagement in self-reflective journal writing. These approaches integrate current advancements in AI and e-learning technologies and building upon previous research (Brooks, 2022; Khellab et al., 2022; Li, 2021; Molin et al., 2020).

Metacognitive theory emphasizes individuals’ awareness of monitoring, evaluating, and regulating their own cognitive processes (Ohtani and Hisasaka, 2018). Grounded in this theoretical framework, the present study adopted a multi-method integrated approach that combined assessment, feedback, and reflection to support the development of metacognitive skills. This approach enabled learners to monitor their practice progress, identify specific problems, and clarify areas for improvement. For example, learners used the audio comparison tool to facilitate self-assessment. As one student remarked, “*I thought I was proficient, but the recording always reveals new problems*” (LY-6).

Moreover, feedback from the AI large language model allowed learners to seek advice on how to address issues in their own practice and optimize their learning strategies (Yuan, 2024). In parallel, the integration of reflective journal writing encouraged learners to actively evaluate their learning processes (Brown, 1987; Müller and Seufert, 2018). The use of reflective journals also stimulated internal reflection and helped learners internalize external feedback, ultimately contributing to metacognitive development (Kuiper, 2002; Moore, 2018).

Based on previous research and the findings of this study, it is proposed that the assessment- feedback–reflection–practice cycle should serve as a key model for enhancing metacognition (Karaoglan Yilmaz, 2022; McPherson et al., 2022; Wu et al., 2020). This study innovatively combines this model with AI and e-learning tools, thereafter applying it to vocal music training.

### 4.3 The implications of AI for vocal music education

Previous research has shown that the use of audio recordings for vocal music self-practice is effective (Hoppe et al., 2006; Mo et al., 2016). In this study, the AI language model was used to generate programming code, which was then used to develop a software tool for audio comparison during singing practice. Encouragingly, tools needed for music teaching can now be independently created and implemented by teachers using AI; tasks that previously required professional programming expertise are now accessible to non-experts.

Traditionally, music learning has primarily relied on face-to-face instruction and timely teacher feedback (Zhang and Leung, 2023). However, in the absence of such direct guidance, students often struggle to identify effective practice strategies independently (McPherson et al., 2019; Mieder and Bugos, 2017). In this study, when students practiced with the assistance of AI-generated feedback, they received more detailed explanations that potentially enhanced their understanding of vocal techniques. Specifically, AI can support instructors by providing timely responses to students’ questions. As one participant noted: “*I always feel that I cannot hit the high notes when I’m practicing. I asked the AI, ‘How do I train my voice to sing high notes?’ The AI told me to first master chest-abdominal joint breathing, then do more training in the middle register, and finally gradually extend upward while paying attention to mouth opening*.” (ZD-3).

However, relying solely on AI to provide feedback is insufficient for improving metacognition. Research has shown that it is difficult to form a mechanism if just feedback is provided, without deep reflection from participants (Wu et al., 2020). In this study, the use of reflective journals played an important role as an “internaliser,” enabling students to repeatedly review their learning process and gradually develop the ability to reflect.

It is important to note that AI-generated dialogue has raised concerns, primarily because the content it produces typically reflects the average patterns of its training data (Dash and Agres, 2024; Yuan, 2024). Given the significant individual differences among music learners, AI-generated feedback may lack the personalization necessary to address specific learner needs (as noted by vocal instructor TW). Therefore, integrating AI-generated feedback with personalized, real-time feedback from teachers is essential to ensure that students receive guidance tailored to their unique learning profiles.

### 4.4 Limitations

For the purposes of this study, an experimental research method was adopted. To ensure the effectiveness of the AI-assisted training, participants were randomly assigned to either the experimental group or the control group, with efforts made to ensure that both groups had as similar backgrounds as possible. However, considering that the control group did not receive active intervention, ethical concerns may arise. To address this, both groups were provided with the same vocal training content, and the control group was scheduled to receive the AI-assisted intervention after the study concluded.

As the experimental group received multiple forms of intervention, including an audio comparison tool, interactive feedback from generative AI, self-reflection journaling, and instructor-provided feedback. Therefore, the improvement in metacognitive development is likely the result of a combined intervention. However, it remains unclear which specific component contributed most significantly to this improvement. Future research could employ variable-control or group-based experimental designs to isolate and examine the individual effects of each intervention, thereby offering a more precise understanding of their respective roles in fostering metacognition.

It is important to note that, at the time of writing, the audio comparison tool developed in this study was still in the testing phase and may require further refinement for wider applications in the future.

## 5 Conclusion

This study aimed to examine the effectiveness of integrating large language models (AI) and e-learning tools into vocal music training, with a particular focus on the impact of feedback- and reflection-based interventions on the metacognitive abilities and singing performance of pre-service teachers. The results indicated that the experimental group showed a significant improvement in metacognitive ability after the training, with a significant interaction effect observed between group and time compared to the control group. Although both the experimental and control groups demonstrated gains in singing performance, no significant interaction effect was found. Additionally, the study revealed that metacognitive levels did not significantly predict singing performance.

The contribution of this study lies in its investigation of effective strategies to enhance learners’ metacognitive abilities through vocal music training. Grounded in the theoretical foundations of feedback and reflection, this research developed a practically applicable instructional intervention designed to promote metacognitive development. It introduces a cyclical metacognitive training model “assessment–feedback–reflection–practice,” which provides both theoretical support and a practical framework for advancing metacognition in music education. This model may be further extended and applied across disciplines to improve teaching and learning outcomes.

## Figures and Tables

**Figure A1:**
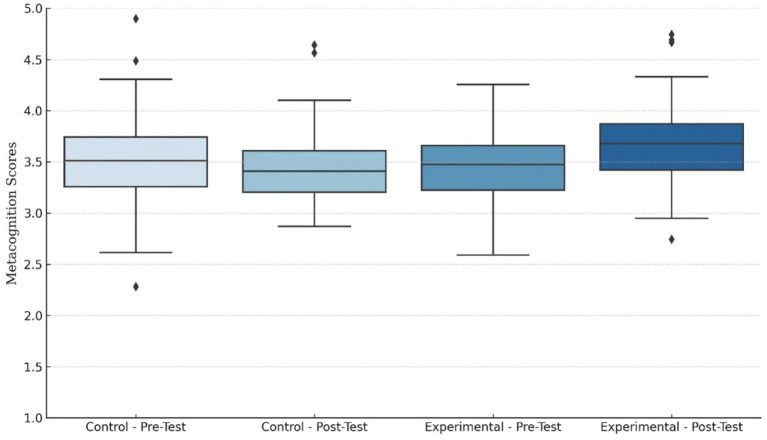
Box plot of metacognition scores for the control and experimental groups at pre-test and post-test. Boxes represent the interquartile range (IQR), and lines within boxes indicate medians. Whiskers extend to 1.5 × IQR; dots represent outliers.

**Figure A2:**
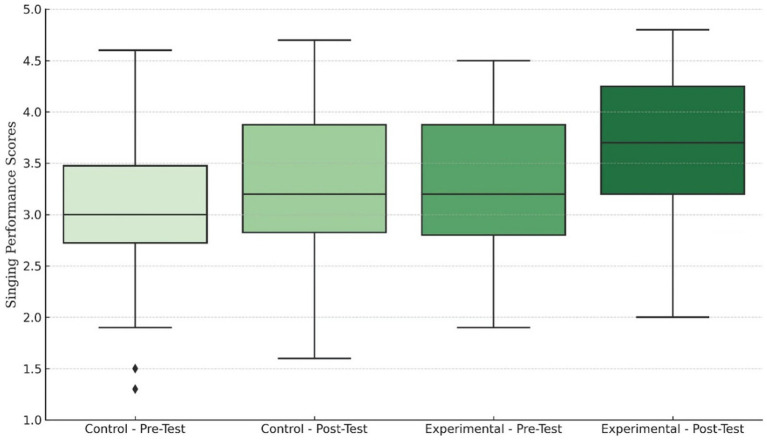
Box plot of singing performance scores for the control and experimental groups at pre-test and post-test. Boxes represent the interquartile range (IQR), and lines within boxes indicate medians. Whiskers extend to 1.5 × IQR; dots represent outliers.

## Data Availability

The original contributions presented in the study are included in the article/supplementary material, further inquiries can be directed to the corresponding author.
